# Seria a Revascularização Completa Verdadeiramente Superior à ICP apenas da Lesão Culpada em Pacientes que Apresentam Infarto Agudo do Miocárdio com Supradesnivelamento do Segmento ST?

**DOI:** 10.36660/abc.20200640

**Published:** 2020-08-19

**Authors:** Christina Grüne de Souza e Silva

**Affiliations:** 1 Clínica de Medicina do Exercício Rio de Janeiro RJ Brasil Clínica de Medicina do Exercício – CLINIMEX, Rio de Janeiro, RJ - Brasil

**Keywords:** Doença Arterial Coronariana, Infarto Agudo do Miocárdio com Supradesnivelamento do Segmento ST, Revascularização do Miocárdio, Intervenção Coronariana Percutânea

A intervenção coronariana percutânea primária (ICP) é uma terapia padrão para pacientes com infarto agudo do miocárdio com supradesnivelamento do segmento ST (IAMCSST) e seu objetivo é restaurar o fluxo sanguíneo para a artéria coronária considerada causadora do infarto do miocárdio (conhecida como artéria culpada). Em até metade desses pacientes, estenoses importantes em uma ou mais artérias coronárias que não são responsáveis pelo infarto do miocárdio (lesões não-culpadas) também podem ser vistas durante a angiografia-índice.^1^ Como os pacientes com IAMCSST e doença arterial coronariana (DAC) multiarterial têm piores resultados clínicos em comparação com pacientes com doença uniarterial, questionou-se se o tratamento por ICP de todas as lesões não-culpadas significativas após a ICP primária (revascularização completa) poderia melhorar o prognóstico.

Uma série de ensaios clínicos randomizados (ECR) abordaram esse tópico, comparando os resultados de pacientes com IAMCSST e DAC multiarterial que foram submetidos a revascularização completa *versus* tratamento por ICP somente da lesão culpada (revascularização incompleta). Anteriormente, ECRs^2 - 4^ de tamanho intermediário mostraram que a revascularização completa é segura e reduz o risco de desfechos compostos, com resultados impulsionados predominantemente pela diminuição do risco de revascularização subsequente. Recentemente, o estudo COMPLETE ( *Complete versus Culprit-Only Revascularization Strategies to Treat Multivessel Disease after Early Percutaneous Coronary Intervention [PCI] for STEMI* ),^5^ um ECR maior, mostrou que o risco do desfecho composto por morte cardiovascular ou infarto do miocárdio recorrente foi menor no grupo de revascularização completa do que no grupo de ICP apenas da lesão culpada em pacientes com IAMCSST, com esse benefício tendo sido impulsionado por uma redução de novo infarto. Além disso, na maior meta-análise de ECRs realizada até o momento abordando este tópico,^6^ a revascularização completa foi associada a uma redução da mortalidade cardiovascular em comparação com a ICP apenas da lesão culpada em pacientes com IAMCSST e DAC multiarterial sem choque cardiogênico à apresentação ( *odds ratio* , 0,69; intervalo de confiança de 95% [IC95%], 0,48-0,99; p = 0,05).

Entretanto, para fornecer uma base de evidências balanceada para a tomada de decisão clínica, os resultados de estudos observacionais devem complementar aqueles obtidos no ECR. Embora seja geralmente aceito que os ECRs são o “padrão ouro” para avaliação de terapias médicas, eles tendem a avaliar intervenções em condições ideais em populações altamente selecionadas, limitando a generalização de seus resultados à prática clínica.

Nesta edição dos Arquivos Brasileiros de Cardiologia, Cadore et al.,^7^ apresentam os resultados de um estudo observacional conduzido em dois hospitais brasileiros comparando a revascularização completa *versus* ICP apenas da lesão culpada em pacientes com IAMCSST e DAC multiarterial. De um total de 85 pacientes que tinham lesões não-culpadas com estenose de pelo menos 70% na estimativa visual (72% do sexo masculino, média de idade de 62 anos), 58 pacientes (68%) foram submetidos à revascularização completa. No grupo de revascularização completa, a minoria dos pacientes (10%) foi submetida à ICP da lesão culpada durante o procedimento índice de ICP para IAMCSST, enquanto 52 pacientes (90%) foram submetidos à revascularização estadiada (isto é, ICP durante um procedimento separado do procedimento de ICP índice para IAMCSST), com tempo médio entre os procedimentos de 13 dias. Após um ano de seguimento, 8% dos pacientes morreram. O desfecho primário composto (mortalidade cardiovascular, novo infarto do miocárdio, angina recorrente) ocorreu em seis pacientes (10%) no grupo de revascularização completa, em comparação com dez pacientes (37%) no grupo de ICP apenas da lesão culpada ( *odds ratio* , 5,1; IC95%, 1,6-16,1; p = 0,005). Morte por causa cardiovascular ocorreu em dois pacientes (3%) submetidos à revascularização completa em comparação com cinco pacientes (19%) submetidos à ICP apenas da lesão culpada ( *odds ratio* , 6,4; IC95%, 1,2-35,3). Acidente vascular cerebral, parada cardíaca não fatal, sangramento grave ou revascularização subsequente (o desfecho secundário composto) ocorreu em três casos (5%) no grupo de revascularização completa em comparação com seis casos (22%) no grupo de ICP apenas da lesão culpada ( *odds ratio* , 5,2; IC95%, 1,2-22,9; p = 0,022); entretanto, essa diferença não foi significativa após o ajuste para possíveis confundidores.

Embora o estudo acima mencionado forneça resultados otimistas que favorecem a revascularização completa, estudos observacionais maiores que abordaram a questão discutida mostraram resultados conflitantes. Ao analisar os dados do *National Cardiovascular Data Registry* , Cavender et al.^8^ verificaram que as taxas gerais de mortalidade hospitalar eram maiores em pacientes submetidos a revascularização completa (7,9% vs. 5,1%, p <0,01), mesmo em pacientes com choque cardiogênico. Da maneira similar, a análise do registro EUROTRANSFER^9^ mostrou que os pacientes submetidos à ICP de lesão não-culpada apresentavam maior risco de morte em 30 dias e 1 ano em comparação aos pacientes com ICP apenas da lesão culpada, embora essa diferença na mortalidade não fosse mais significativa após ajuste para potenciais covariáveis. Por outro lado, Dimitriu-Leen et al.,^10^ observaram que a taxa de mortalidade em 1 ano de seguimento foi significativamente maior nos pacientes tratados com revascularização incompleta em comparação aos pacientes submetidos à revascularização completa (9,8% vs. 4,3%, respectivamente, p = 0,02). Entretanto, após a análise por regressão de Cox multivariada, a revascularização incompleta não mostrou uma associação independente com o aumento da mortalidade por todas as causas.

Considerando os resultados apresentados acima, poderíamos concluir que pacientes com IAMCSST e DAC multiarterial devem ser submetidos a revascularização completa? Antes de responder a essa pergunta, alguns problemas encontrados nos estudos que abordam esse tópico devem ser ressaltados. Primeiro, há uma grande heterogeneidade nos protocolos adotados entre os ensaios clínicos randomizados e estudos observacionais que comparam a revascularização completa *versus* a ICP apenas da lesão culpada, principalmente em relação ao momento da ICP das lesões não-culpadas – durante o procedimento de ICP índice ou como revascularização estadiada, e os critérios utilizados para definir estenose significativa - 50% ou 70% determinados visualmente ou guiados pela medida da reserva de fluxo fracionada, o que dificulta a comparabilidade entre os resultados relatados. Segundo, principalmente em relação ao ECR, deve-se levar em consideração a possibilidade de viés de publicação, quando estudos com resultados estatisticamente significativos têm um aumento da probabilidade de serem publicados, neste caso, favorecendo a revascularização completa. Terceiro, particularmente em relação ao artigo publicado nesta edição da revista Arquivos Brasileiros de Cardiologia, estudos com pequena amostra apresentam maior risco de certos tipos de viés, o que pode alterar significativamente seus achados, favorecendo uma ou outra estratégia e, portanto, estudos observacionais maiores ainda são necessários para confirmar os achados de Cadore et al.^7^ Além disso, embora alguns poucos estudos tenham mostrado diferenças em desfechos duros, como infarto do miocárdio e morte cardiovascular, favorecendo a revascularização completa, a maioria não mostra diferença na mortalidade por todas as causas, o que pode sugerir que outras causas de morte potencialmente associadas aos procedimentos de ICP, como infecção, podem não ter sido consideradas.

Finalmente, o tratamento de lesões não-culpadas com ICP no IAMCSST poderia ser discutido em um contexto mais amplo, com base no impacto da revascularização percutânea de lesões estáveis. Evidências recentes fornecidas pelo estudo ISCHEMIA ( *International Study of Comparative Health Effectiveness With Medical and Invasive Approaches* )^11^ mostraram que em pacientes com doença coronariana estável que apresentavam isquemia moderada ou grave, uma estratégia invasiva inicial, em comparação com uma estratégia conservadora inicial, não reduziu as taxas do desfecho primário composto morte cardiovascular, infarto do miocárdio ou hospitalização por angina instável, insuficiência cardíaca ou parada cardíaca não fatal. Portanto, embora pacientes e médicos se sintam mais à vontade com a revascularização de todas as estenoses coronárias do que com a terapia medicamentosa, são necessários mais dados dos ECRs e dos estudos observacionais para avaliar se a revascularização completa fornece benefícios adicionais sobre a ICP apenas da lesão culpada em pacientes com IAMCSST e DAC multiarterial. Por enquanto, uma abordagem razoável deve incorporar o julgamento clínico e qualquer benefício da revascularização de lesões em artérias não-culpadas deve ser contrabalançado por possíveis desvantagens dos procedimentos de ICP adicionais.
