# Supplementary material for: Seria a Revascularização Completa Verdadeiramente Superior à ICP apenas da Lesão Culpada em Pacientes que Apresentam Infarto Agudo do Miocárdio com Supradesnivelamento do Segmento ST?
Source: Arq Bras Cardiol. 2020 Aug 19;115(2):238–40. [Article in Portuguese] doi: 10.36660/abc.20200640 (PMC8384295; doi:10.36660/abc.20200640)
Supplement: Translation

# Translation: Is Complete Revascularization Truly Superior to Culprit-Lesion-Only PCI in Patients Presenting with ST-segment Elevation Myocardial Infarction?

**Authors:** Christina Grüne de Souza e Silva

**Affiliations:** 1 Brazil Clínica de Medicina do Exercício – CLINIMEX, Rio de Janeiro, RJ - Brazil

**Keywords:** Coronary Artery Disease, ST Elevation Myocardial Infarction, Myocardial Revascularization, Percutaneous Coronary Intervention

Primary percutaneous coronary intervention (PCI) is a standard therapy for patients with acute ST-segment elevation myocardial infarction (STEMI), and its goal is to restore blood flow to the coronary artery that is judged to be causing the myocardial infarction (known as the culprit artery). In up to half of such patients, major stenoses in one or more coronary arteries that are not responsible for the myocardial infarction (nonculprit lesions) may also be seen during the index angiography.^1^ Since patients with acute STEMI and multivessel coronary artery disease (CAD) have worse clinical outcomes compared with patients with single-vessel disease, it has been questioned if PCI treatment of all significant nonculprit lesions following primary PCI (complete revascularization) could improve prognosis.

A number of randomized clinical trials (RCT) have addressed this topic by comparing outcomes of patients with STEMI and multivessel CAD who underwent complete revascularization versus treatment of the culprit-lesion-only PCI (incomplete revascularization). Previously, intermediate-sized RCT^2 - 4^ have shown that complete revascularization is safe and reduces the risk of composite outcomes, with results driven predominantly by the decreased risk of subsequent revascularization. Recently, the COMPLETE (Complete versus Culprit-Only Revascularization Strategies to Treat Multivessel Disease after Early Percutaneous Coronary Intervention [PCI] for STEMI) trial,^5^ a larger RCT, showed that the risk of the composite outcome death from cardiovascular causes or recurrent myocardial infarction was lower in the complete revascularization group than in the culprit-lesion-only PCI group in patients presenting with STEMI, this benefit been driven by a reduction in new myocardial infarction. Moreover, in the largest meta-analysis of RCT performed to date addressing this topic,^6^ complete revascularization was associated with a reduction in cardiovascular mortality compared with culprit-lesion-only PCI in patients with STEMI and multivessel CAD without cardiogenic shock at presentation (odds ratio, 0.69; 95% confidence interval [CI], 0.48-0.99; p=0.05).

However, in order to provide balanced evidence base for clinical decision making, results from observational studies should complement the ones obtained from RCT. Although it is generally accepted that RCT are the “gold standard” for evaluation of medical therapies, these tend to evaluate interventions under ideal conditions among highly selected populations, limiting its provided evidence generalizability to clinical practice.

In this issue of the journal *Arquivos Brasileiros de Cardiologia* , Cadore et al.^7^ presents results from an observational study conducted in two Brazilian hospitals comparing complete revascularization vs. culprit-lesion-only PCI in patients with STEMI and multivessel CAD. From a total of 85 patients who had nonculprit lesions with stenosis of at least 70% on visual estimation (72% male, mean age of 62 years), 58 patients (68%) underwent complete revascularization. In the complete revascularization group, the minority of patients (10%) underwent nonculprit lesion PCI during the index PCI procedure for STEMI, while 52 patients (90%) underwent staged revascularization (i.e., PCI during a procedure separate from the index PCI procedure for STEMI), with a mean time between procedures of 13 days. After one year of follow-up, 8% of patients died. Primary composite outcome (cardiovascular mortality, new myocardial infarction, recurrent angina) had occurred in six patients (10%) in the complete revascularization group as compared with ten patients (37%) in the culprit-lesion-only PCI group (odds ratio, 5.1; 95% CI, 1.6 to 16.1; p=0.005). Death due to cardiovascular causes occurred in two patients (3%) who underwent complete revascularization compared to five patients (19%) who underwent culprit-lesion-only PCI (odds ratio, 6.4; 95% CI, 1.2 to 35.3). Stroke, non-fatal cardiac arrest, major bleeding, or subsequent revascularization (the second composite outcome), occurred in three (5%) in the complete revascularization group as compared with six patients (22%) in the culprit-lesion-only PCI group (odds ratio, 5.2; 95% CI, 1.2 to 22.9; p=0.022); however, this difference was not significant after adjusting for potential cofounders.

Although the aforementioned study provides optimistic outcomes favoring complete revascularization, larger observational studies that addressed the discussed issue have shown conflicting results. When analyzing data from the National Cardiovascular Data Registry, Cavender et al.^8^ found that the overall in-hospital mortality rates were greater in patients undergoing complete revascularization (7.9% vs. 5.1%, p<0.01), even for patients presenting with cardiogenic shock. Similarly, the analysis of the EUROTRANSFER Registry^9^ showed that patients who underwent nonculprit lesion PCI were at higher risk of 30-day and 1-year death compared to patients with culprit-lesion-only PCI, although this difference in mortality was no longer significant after adjustment for potential covariates. In contrast, Dimitriu-Leen et al.^10^ found that the mortality rate at 1 year of follow-up was significantly higher for patients who were treated with incomplete revascularization compared with patients who underwent complete revascularization (9.8% vs 4.3%, respectively, p=0.02). However, after performing multivariate Cox regression analysis, incomplete revascularization was not independently associated with increased all-cause mortality.

In view of the results presented above, could we draw the conclusion that patients presenting with STEMI and multivessel CAD should undergo complete revascularization? Before answering this question, some issues found in the studies addressing this topic should be underscored. First, there is a great heterogeneity in the adopted protocols between both RCT and observational studies that compare complete revascularization vs. culprit-lesion-only PCI, mostly regarding to the timing of nonculprit lesion PCI - during the index PCI procedure or as staged revascularization, and the criteria used to define significant stenosis - 50% or 70% visually determined or guided by fractional flow reserve measurement, which hampers the comparability between the reported results. Second, especially regarding to RCT, it should be taken into account the possibility of publication bias, when studies with statistically significant results have increased likelihood of being published, in this case, favoring complete revascularization. Third, particularly regarding to the published article in this issue of the *Arquivos Brasileiros de Cardiologia* journal, small sample studies are of greater risk of certain types of bias that can significantly alter their findings favoring one or another strategy, and, thus, larger observational studies are further needed to confirm Cadore et al.^7^ findings. Moreover, although few studies have shown differences in hard outcomes such as myocardial infarction and cardiovascular death favoring complete revascularization, the majority does not show any difference in all-cause mortality, which might suggest that other causes of death potentially associated with PCI procedures, such as infection, might not have been considered.

Finally, whether to treat nonculprit lesions with PCI in STEMI could be discussed in a larger context, based on the impact of stenting stable lesions. Recent evidence provided by the ISCHEMIA (International Study of Comparative Health Effectiveness With Medical and Invasive Approaches) trial^11^ showed that among patients with stable coronary disease who had moderate or severe ischemia, an initial invasive strategy, as compared with an initial conservative strategy, did not reduce the rates of the primary composite outcome death from cardiovascular causes, myocardial infarction, or hospitalization for unstable angina, heart failure, or resuscitated cardiac arrest. Therefore, although patients and clinicians are often more comfortable with revascularization of all suitable coronary stenosis rather than medical therapy, more data from both RCT and observational studies is needed to evaluate if complete revascularization provides additional benefit over culprit-lesion-only PCI in patients with STEMI and multivessel CAD. For now, a reasonable approach should incorporate clinical judgment, and any benefit of revascularization of lesions in nonculprit arteries should be counterbalanced by potential disadvantages of additional PCI procedures.
